# Assessing the Clinical Relevance of Blood Soluble Triggering Receptor Expressed on Myeloid Cells 2 in Neurological Diseases

**DOI:** 10.1111/ejn.70690

**Published:** 2026-09-10

**Authors:** Luisa Agnello, Caterina Maria Gambino, Giuseppe Salemi, Vincenzo Di Stefano, Tommaso Piccoli, Anna Masucci, Sabrina Novara, Anna Maria Ciaccio, Fabio Del Ben, Daniela Capello, Marcello Ciaccio

**Affiliations:** ^1^ Department of Biomedicine, Neurosciences and Advanced Diagnostics, Institute of Clinical Biochemistry, Clinical Molecular Medicine, and Clinical Laboratory Medicine University of Palermo Palermo Italy; ^2^ Department of Laboratory Medicine University Hospital Paolo Giaccone Palermo Italy; ^3^ Department of Biomedicine, Neurosciences and Advanced Diagnostics, Unit of Neurology University of Palermo Palermo Italy; ^4^ Department of Health Promotion, Mother and Child Care, Internal Medicine and Medical Specialities University of Palermo Palermo Italy; ^5^ Immunopathology and Cancer Biomarkers Centro di Riferimento Oncologico (CRO)‐IRCCS Aviano Italy; ^6^ UPO Biobank University of Piemonte Orientale Novara Italy

**Keywords:** Alzheimer's disease, ATTR polyneuropathy, mild cognitive impairment, multiple sclerosis, sTREM2

## Abstract

Triggering receptor expressed on myeloid cells 2 (TREM2) is a key regulator of microglial and peripheral myeloid cell function, with its soluble form (sTREM2) emerging as a biomarker of neuroinflammation. Although cerebrospinal fluid (CSF) sTREM2 is consistently elevated in neurodegenerative diseases, the clinical relevance of blood sTREM2 remains unclear. We performed a retrospective observational study including 547 individuals: Alzheimer's disease (ad, *n* = 142), mild cognitive impairment (MCI, *n* = 42), multiple sclerosis (MS, *n* = 105), hereditary transthyretin amyloidosis with polyneuropathy (ATTR‐PN, *n* = 23), asymptomatic ATTR mutation carriers (*n* = 25), and controls (*n* = 210). Plasma sTREM2 concentrations were measured using Lumipulse automated chemiluminescent enzyme immunoassay platform (Fujirebio, Tokyo, Japan). Age‐adjusted multivariate analysis was performed to compare disease groups with controls. In controls, plasma sTREM2 levels increased with age, with a significant breakpoint at 68 years, after which the rate of increase markedly accelerated. Across diagnostic groups, ad, MCI, and MS patients exhibited significantly lower plasma sTREM2 levels (11%–18% reduction) compared to controls (*p* < 0.05), independent of age. No significant differences were observed in ATTR‐PN patients or asymptomatic carriers. Age remained a strong positive predictor of sTREM2 levels across all groups. In conclusions, plasma sTREM2 levels are reduced in ad, MCI, and MS despite known elevations in CSF, indicating a divergence between peripheral and central TREM2 biology. The lack of alteration in ATTR‐PN suggests tissue‐specific regulation of sTREM2. These findings highlight that blood sTREM2 does not directly mirror central nervous system microglial activation but may reflect peripheral immune dynamics, warranting further investigation into its role as a biomarker of systemic immune dysfunction in neurodegenerative diseases.

## Graphical abstract

Blood sTREM2 is emerging as a potential biomarker of neuroinflammation, yet its biological significance remains poorly understood. In this study, plasma sTREM2 was quantified in 547 individuals across Alzheimer's disease, mild cognitive impairment, multiple sclerosis, ATTR polyneuropathy, asymptomatic ATTR mutation carriers, and healthy controls. Plasma sTREM2 increased with age, with an accelerated rise after 68 years, but was selectively reduced in Alzheimer's disease, mild cognitive impairment and multiple sclerosis independently of age. In contrast, no changes were observed in ATTR polyneuropathy. These findings suggest that plasma sTREM2 does not simply reflect central microglial activation, but instead captures disease‐specific alterations in peripheral TREM2 biology.

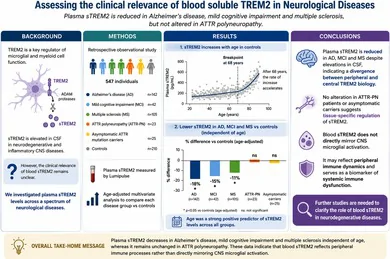

**Abbreviations**
ad
: Alzheimer's disease
ADAM: A Disintegrin and Metalloproteinase
ATTR‐PN: hereditary transthyretin amyloidosis with polyneuropathy
CI: confidence interval
CNS: central nervous system
CSF: cerebrospinal fluid
EDTA: ethylenediaminetetraacetic acid
GFAP: glial fibrillary acidic protein
IQR: interquartile range
LoD: limit of detection
LoQ: limit of quantification
MCI: mild cognitive impairment
MRI: magnetic resonance imaging
MS: multiple sclerosis
OLS: ordinary least squares
PET: positron emission tomography
RRMS: relapsing–remitting multiple sclerosis
sTREM2: soluble Triggering Receptor Expressed on Myeloid Cells 2
TREM2: Triggering Receptor Expressed on Myeloid Cells 2
TTR: transthyretin

## 1 Introduction

Neuroinflammation is a central pathological hallmark of various neurodegenerative and central nervous system (CNS) disorders, including Alzheimer's disease (ad), multiple sclerosis (MS), and other neurological conditions (Zhang et al. 2023). Microglia, the resident immune cells of the brain, play pivotal roles in regulating neuronal function under both physiological and pathological conditions, orchestrating inflammatory responses, phagocytosis, and tissue repair (Qu and Li 2023). The triggering receptor expressed on myeloid cells 2 (TREM2) is a transmembrane immune receptor predominantly expressed by microglia in the CNS, where it serves as a key regulator of microglial activation, survival, metabolism, and phenotypic switching (Zhang et al. 2023; Pocock et al. 2024). Genome‐wide association studies have identified loss‐of‐function variants in *TREM2* gene that are associated with a two‐ to fourfold increased risk of late‐onset ad, underscoring its critical role in neurodegeneration (Ayyubova 2023; Carmona et al. 2018). TREM2 undergoes proteolytic cleavage by ADAM family proteases, releasing a soluble fragment (sTREM2) into biological fluids, including cerebrospinal fluid (CSF) and blood (Morenas‐Rodríguez et al. 2022). CSF sTREM2 has emerged as a promising biomarker of microglial activation and TREM2 signaling in neurodegenerative diseases. Cross‐sectional and longitudinal studies have consistently demonstrated elevated CSF sTREM2 levels in preclinical and early symptomatic stages of ad, with peak concentrations observed at the mild cognitive impairment (MCI) stage (Carmona et al. 2018; Schlepckow et al. 2023). Additionally, increased baseline CSF sTREM2 levels have been associated with slower hippocampal atrophy, improved cognitive trajectories, and reduced risk of MCI‐to‐ad conversion, suggesting a protective role for TREM2‐mediated microglial responses in early disease phases (Zhao et al. 2022). Similarly, CSF sTREM2 is significantly elevated in patients with relapsing–remitting, primary progressive, and secondary progressive MS compared to non‐inflammatory controls, reflecting CNS inflammation and microglial activation (Piccio et al. 2008; Öhrfelt et al. 2016). Despite the robust evidence supporting CSF sTREM2 as a biomarker of CNS pathology, its clinical utility is limited by the invasive nature of lumbar puncture. Consequently, there has been considerable interest in evaluating blood‐based sTREM2 as a more accessible alternative. However, the relationship between peripheral and central sTREM2 remains controversial. Some studies have reported a correlation between plasma and CSF sTREM2 levels, whereas others have found an inverse or no significant relationship (Zhao et al. 2022; Park et al. 2021). The poor correlation between peripheral and central inflammatory markers is a recognized limitation of blood‐based biomarkers for neurological diseases, as peripheral levels may not accurately reflect CNS pathology (Gigase et al. 2023). Notably, however, a poor correlation between blood and CSF levels does not preclude clinical utility for all biomarkers. Glial fibrillary acidic protein (GFAP) provides a compelling example. Despite plasma GFAP showing greater magnitude changes than CSF GFAP across the ad continuum, and plasma GFAP more accurately discriminating amyloid‐β‐positive from amyloid‐β‐negative individuals than its CSF counterpart, the correlation between plasma and CSF GFAP is only modest (Benedet et al. 2021; Pereira et al. 2021). Plasma GFAP elevations appear a decade before expected symptom onset, predict subsequent development of ad in patients with MCI, and are associated with cognitive decline and neurodegeneration, establishing it as a clinically valuable blood‐based biomarker despite its imperfect relationship with CSF levels (Teunissen et al. 2022; Chatterjee et al. 2023; Shen et al. 2023). This observation raises the question of whether blood sTREM2, despite its poor correlation with CSF sTREM2, might still provide clinically relevant information in specific disease contexts.

The clinical relevance of blood sTREM2 in neurological diseases beyond ad and MS, such as hereditary transthyretin amyloidosis with polyneuropathy (ATTR‐PN), remains unexplored. ATTR‐PN is a progressive systemic disorder characterized by extracellular deposition of misfolded transthyretin protein, leading to peripheral neuropathy and multiorgan involvement. Pathological studies have demonstrated robust infiltration of pro‐inflammatory macrophages in peripheral nerves of ATTR‐PN patients, with activation of the NLRP3 inflammasome and disruption of the blood–nerve barrier contributing to nerve fiber loss (Chao et al. 2025). Importantly, TREM2 is not exclusively expressed by CNS microglia but is also expressed on peripheral myeloid cells, including tissue macrophages, monocytes, and dendritic cells, where it regulates activation, polarization, phagocytosis, and cytokine secretion (Medd 2025; Colonna 2023). Furthermore, TREM2 is expressed in Schwann cells, the principal glial cells of the peripheral nervous system, where it plays a critical role in energy metabolism and nerve regeneration; TREM2 deficiency exacerbates peripheral neurological deficits and impairs axonal regeneration in experimental models of peripheral neuropathy (Zhang et al. 2024). Given that sTREM2 is released into the extracellular space following proteolytic cleavage by ADAM10/17 and meprin β from both microglia and peripheral macrophages, blood sTREM2 levels may reflect not only CNS microglial activation but also peripheral macrophage and Schwann cell activity (Carmona et al. 2018; Berner et al. 2020). Whether peripheral sTREM2 reflects disease activity or macrophage/glial activation in ATTR‐PN is unknown, providing a rationale for investigating blood sTREM2 as a potential biomarker in this condition.

The aim of this study was to assess the clinical relevance of blood sTREM2 across a spectrum of neurological diseases, including ad, MCI, MS, and ATTR‐PN, compared to individuals from the general population, adjusting for confounders.

## 2 Methods

### 2.1 Study Population

This is a retrospective, cross‐sectional, case–control observational study performed at the University Hospital “Paolo Giaccone” of Palermo (Italy). Plasma sTREM2 was measured at a single time point in each participant. The study population consisted of five clinically defined patient groups: ad, MCI, MS, ATTR‐PN, and asymptomatic ATTR mutation carriers. NCS were collected from the Novara subgroup. All neurological diagnoses were made by expert neurologists according to the criteria detailed below.

### 2.2 Inclusion and Exclusion Criteria

Inclusion criteria were confirmed diagnosis of ad, MCI, MS, ATTR‐PN, or asymptomatic ATTR mutation carriers; availability of a plasma sample of adequate volume and quality collected within the study period; minimum age of 18 years. Exclusion criteria were concomitant acute infectious or inflammatory illness at the time of sampling; active malignancy; other neurodegenerative or neuroinflammatory comorbidity; missing key clinical data required for diagnostic classification.

ad diagnosis was established using a combination of clinical criteria and biomarker evidence. Clinically, patients exhibited progressive cognitive decline, primarily in memory and learning, with impairment in at least one additional cognitive domain and no evidence of mixed etiology. Biomarker confirmation included amyloid‐β and phosphorylated tau positivity via CSF analysis and/or PET imaging (Jack et al. 2024).

MCI was diagnosed in individuals with subjective or informant‐reported cognitive decline, who performed at least 1.5 standard deviations below age‐ and education‐adjusted norms in one or more cognitive domains on neuropsychological testing, but did not meet criteria for dementia (i.e., preserved independence in daily activities) (Pichet Binette et al. 2025).

MS diagnosis was based on the 2017 and 2024 McDonald criteria, which require demonstration of dissemination in space (lesions in at least two of five CNS locations: periventricular, cortical/juxtacortical, infratentorial, spinal cord, or optic nerve) and dissemination in time (simultaneous presence of gadolinium‐enhancing and non‐enhancing lesions, or new lesions on follow‐up MRI). CSF‐specific oligoclonal bands and exclusion of alternative diagnoses were also required (Montalban et al. 2025; Jakimovski et al. 2024).

ATTR‐PN diagnosis was established through a combination of genetic testing, tissue biopsy, and clinical evaluation (Adams et al. 2023). Asymptomatic carriers were individuals with a confirmed TTR pathogenic variant who exhibited no symptoms or signs of multisystem involvement at enrolment.

Control samples were collected from the Novara cohort study, a longitudinal population study of individuals from the general population (Cracas et al. 2025).

All individuals signed informed consent. The study was conducted in accordance with the Declaration of Helsinki and approved by the Institutional Review Board of Policlinico “Paolo Giaccone” Palermo (n.2/2026).

### 2.3 Plasma sTREM2 Measurement

All blood samples were obtained at the first clinical visit, before a definitive diagnosis was established and prior to the initiation of any disease‐specific treatment.

Plasma sTREM2 was measured using the Lumipulse G1200 automated chemiluminescent enzyme immunoassay platform (Fujirebio, Tokyo, Japan). Venous blood samples were collected into EDTA‐containing tubes and centrifuged at 2000 × g for 10 min at room temperature within 2 h of collection. Plasma was immediately aliquoted into sterile polypropylene tubes and stored at −80°C until analysis. Samples were thawed only once immediately before analysis, with no prior freeze–thaw cycles, in order to minimize protein degradation and pre‐analytical variability.

The Lumipulse G1200 utilizes a two‐step sandwich immunoassay format with chemiluminescent detection. The assay employs monoclonal antibodies specific for sTREM2, with one antibody immobilized on magnetic beads for capture and a second antibody conjugated to alkaline phosphatase for detection. The automated platform performed all subsequent steps: incubation with capture antibodies, washing steps to remove unbound material, addition of detection antibody, and chemiluminescent substrate reaction. Quality control samples at low, medium, and high concentrations were included in each analytical run to ensure assay performance consistency. This automated assay offers high sensitivity and specificity for sTREM2 detection, with a limit of detection of 2 pg/mL, a limit of quantification (LoQ, at 20% CV) of 2.2 pg/mL, and within‐laboratory precision of 2.9%–6.2%.

### 2.4 Statistical Analysis

Outliers were identified and excluded using the interquartile range (IQR) 1.5 method, removing observations exceeding the range defined by [Q1–1.5 × IQR, Q3 + 1.5 × IQR]. Given the non‐normal, right‐skewed distribution of raw sTREM2 levels, a logarithmic transformation was applied.

To assess potential nonlinear changes in sTREM2 levels relative to age, segmented regression analysis was performed using the *segmented* v 2.2 R package, estimating the breakpoint via an iterative method. The significance of the change in slope was formally verified using Davies' test, which assesses the null hypothesis of a constant slope against the alternative of a change point.

To evaluate differences in plasma sTREM2 levels across diagnostic categories while controlling for age as a continuous confounder, a multivariable linear regression model was fitted. The dependent variable was defined as the log‐transformed sTREM2 concentration ($\text{sTREM}2\_\log=\ln\left(\text{sTREM}2+1\right)$) to meet the distributional assumptions of ordinary least squares (OLS) regression residuals. Diagnostic status was modeled as a categorical predictor, with the control group explicitly set as the reference baseline category. Participant age was included as a continuous covariate to adjust for age‐dependent variations in biomarker concentration. To determine whether the relationship between age and sTREM2 levels varied across diagnostic groups, the interaction term (disease × age) was tested. As the interaction was not statistically significant (*p* > 0.05), a parsimonious additive model was adopted, without the interaction term.

$$\mathrm{Log}\;\left(\text{sTREM2 level}\right)\sim \text{Diagnosis}+\mathrm{Age}\;\left(\text{years}\right)$$

Model goodness‐of‐fit and overall explanatory power were evaluated using the global F‐statistic and the adjusted coefficient of determination (adjusted *R* ^2^). Statistical significance was defined as α=0.05.

All analyses were conducted in R Version 4.5.2. Statistical significance was established at α = 0.05.

## 3 Results

The study population consisted of 547 individuals, including patients with ad (*n* = 142), MCI (*n* = 42), MS (*n* = 105), ATTR (*n* = 23), ATTR carriers (*n* = 25), and controls (*n* = 210) (Table 1). Among MS patients, 84% had relapse remitting form, 6% primary progressive, 2% progressive‐relapsing, and 6% secondary progressive. Table 2 summarizes the demographic and clinical characteristics of the study population.

**TABLE 1:** Characteristics of the study population.

	Nr	Age, years, median [IQR]	Male (%)	sTREM2, pg/mL, median [IQR]
ad	140	70 [11]	50.7%	2306 [1153]
ATTR carriers	19	54 [19]	63.2%	1712 [855]
ATTR patients	22	68 [7]	50%	2517 [1860]
MCI	42	75 [12]	40.5%	2320 [1277]
MS	105	42 [20]	21%	1531 [895]
Controls	210	68 [25]	56.2%	2452 [1658]

Abbreviations: ad, Alzheimer's disease; ATTR, hereditary transthyretin amyloidosis; MCI, mild cognitive impairment; MS, multiple sclerosis.

**TABLE 2:** Summary of linear model coefficients.

	Estimate	Std error	*p*	Significant
ad	−0.109	0.052	0.037	*
ATTR carriers	−0.204	0.113	0.072	ns
ATTR patients	0.037	0.105	0.724	ns
MCI	−0.176	0.081	0.029	*
MS	−0.146	0.064	0.024	*
Age	0.015	0.002	< 0.001	***

Abbreviations: ad, Alzheimer's disease; ATTR, hereditary transthyretin amyloidosis; MCI, mild cognitive impairment; MS, multiple sclerosis; ns, not statistically significant.

### 3.1 sTREM2 in Controls

**FIGURE 1:**
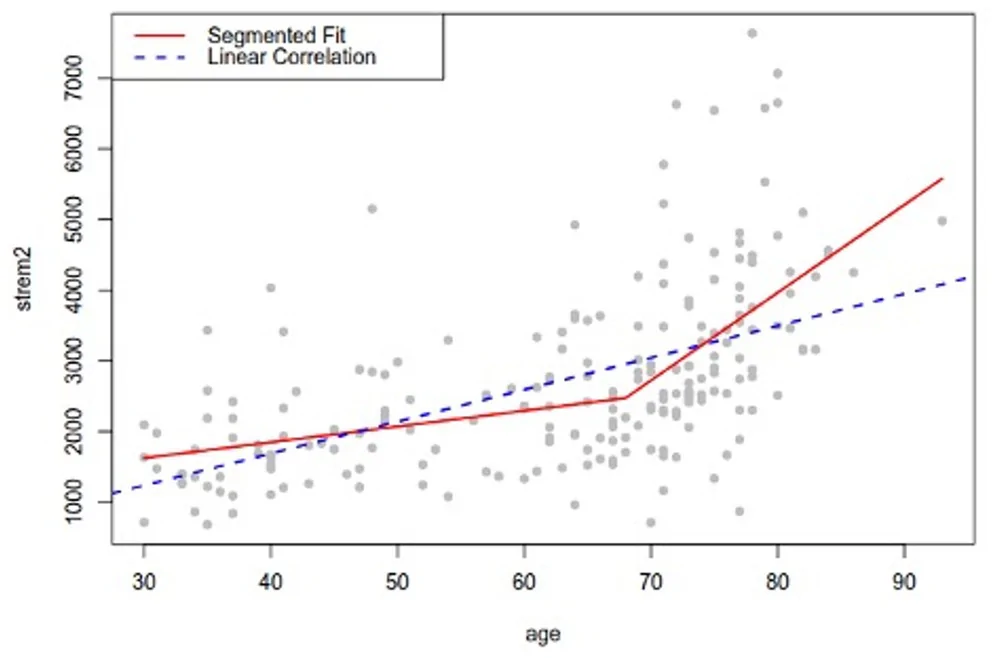
Correlation analysis between plasma sTREM2 levels and age. In red, the segmented (broken line) fit, in blue and dashed, the linear correlation fit.

We first examined the impact of demographic variables on plasma sTREM2 levels in controls (*n* = 210 → 208 after removal of two outliers). Although sex did not significantly influence sTREM2 concentrations (*p* = 0.46), a robust positive correlation was observed between age and plasma sTREM2 levels (ρ Spearman = 0.60, *p* < 0.001) (Figure 1).

To further characterize this relationship (given the nonlinear pattern observed in the scatterplot), we performed a segmented regression analysis (broken‐line model) to identify potential shifts in sTREM2 dynamics across the lifespan (Figure 1). The analysis revealed a significant nonlinear pattern, with a clear breakpoint in sTREM2 accumulation. Specifically, the model identified a significant breakpoint at 68 years (SE = 2.6). The rate of sTREM2 increase was significantly more pronounced after this threshold; the initial slope of 22 pg/mL per year (95% CI, 6–39) increased nearly sixfold to 124 pg/mL per year (95% CI, 80–169) in individuals older than 68. Davies' test confirmed that the change in slope was statistically significant (*p* < 0.001).

To account for potential heteroscedasticity, the analysis was repeated using log‐transformed sTREM2 levels. This model also estimated a breakpoint at 68 years (SE = 3.6). Similarly, the slope increased significantly from 0.01 (95% CI, 0.01–0.02) before the breakpoint to 0.04 (95% CI, 0.02–0.06) thereafter, with Davies' test remaining significant (*p* = 0.016).

These findings suggest that, although sTREM2 levels rise gradually throughout adulthood, there is an accelerated elevation beginning in the seventh decade of life.

### 3.2 STREM2 in Neurological Diseases

We evaluated the association between diagnostic status (Dx) and plasma sTREM2 levels while controlling for age. The dependent variable was log‐transformed (strem2_log). The control group was set as the reference category (baseline).

The model demonstrated a statistically significant explanatory power (*F*_6,531_ = 31.26, *p* < 2.2 × 10–16), accounting for 25.3% of the total variance in sTREM2 levels.

Age emerged as a highly robust predictor of sTREM2 levels. We observed a significant positive linear relationship, indicating an average increase of approximately 1.5% in sTREM2 levels for each additional year of age. Compared to the control group, patients with ad, MCI, and MS exhibited significantly lower sTREM2 levels, with values between −11% and −18%. No statistically significant differences in sTREM2 levels were observed between the control group and ATTR patients or carriers.

## Discussion

This study explored the clinical relevance of plasma sTREM2 across a spectrum of neurological diseases characterized by neuroinflammation and glial activation, including ad, MCI, MS, and ATTR‐PN. Two principal findings emerged. First, plasma sTREM2 increased progressively with age in healthy individuals, with a marked acceleration after approximately 68 years of age. Second, after adjustment for age, patients with ad, MCI, and MS exhibited significantly lower plasma sTREM2 concentrations than healthy controls, whereas no significant differences were observed in ATTR‐PN patients or asymptomatic carriers.

The age‐related increase in plasma sTREM2 represents one of the most relevant observations of this study. Previous investigations have reported a positive association between CSF or circulating sTREM2 and aging (Henjum et al. 2016), suggesting that TREM2 reflects age‐related changes in innate immune activity. Our findings extend these observations by demonstrating that this relationship is nonlinear, with a significant breakpoint occurring at approximately 68 years of age, after which circulating sTREM2 increases more rapidly. This pattern is consistent with the progressive immune remodeling associated with aging, commonly referred to as inflammaging, which is characterized by chronic low‐grade inflammation, accumulation of senescent cells, and altered myeloid cell function (Qu and Li 2023; Pocock et al. 2024). Because TREM2 is expressed by peripheral monocytes, macrophages, and dendritic cells, where it regulates phagocytosis, lipid metabolism, and inflammatory responses (Zhou et al. 2025; Gawish et al. 2015; Muñoz‐García et al. 2025), the observed increase may reflect the physiological adaptation of the innate immune system to aging. From a clinical perspective, these findings indicate that age is a major determinant of plasma sTREM2 concentrations and should be carefully considered when interpreting this biomarker.

The second major finding is that patients with ad, MCI, and MS had significantly lower plasma sTREM2 concentrations than controls, independently from age. Importantly, these differences remained significant after adjustment for age, indicating that they cannot be explained by demographic differences alone. Rather than simply reflecting chronological aging, plasma sTREM2 appears to follow a disease‐specific trajectory in disorders involving CNS pathology. The consistency of this reduction across ad, MCI, and MS suggests that these apparently distinct diseases may share alterations affecting peripheral TREM2 biology.

Another important observation is that this pattern was not found in ATTR‐PN patients or asymptomatic mutation carriers. Although ATTR‐PN is associated with peripheral nerve inflammation and macrophage activation, circulating sTREM2 concentrations were comparable to those of healthy controls. These findings suggest that plasma sTREM2 is not a general biomarker of neurological disease or systemic inflammation but may be selectively altered in disorders characterized by CNS involvement. Whether this reflects differences in tissue compartmentalization, immune activation, or disease biology warrants further investigation.

Our findings also emphasize that plasma and CSF sTREM2 provide distinct biological information. Increased CSF sTREM2 has been consistently reported in ad and MS and is generally interpreted as a biomarker of microglial activation associated with neurodegeneration (Carmona et al. 2018; Schlepckow et al. 2023; Zhao et al. 2022; Azzolini et al. 2022). In contrast, we observed reduced plasma concentrations in the same diseases, supporting previous evidence that circulating sTREM2 is regulated independently from its CSF counterpart (Park et al. 2021). We recently evaluated sTREM2 simultaneously in CSF, serum, and plasma using the same fully automated Lumipulse assay (Agnello et al. 2026), demonstrating only a modest correlation between CSF and blood despite a strong agreement between serum and plasma measurements. Therefore, plasma sTREM2 cannot be considered a direct surrogate of CNS microglial activation. Several mechanisms, including differences in TREM2 shedding, compartment‐specific regulation, or redistribution of TREM2‐expressing myeloid cells, have been proposed in the literature (Berner et al. 2020; Zhou et al. 2025; Rossi et al. 2021; Zierfuss et al. 2024; Zang et al. 2022; Bekris et al. 2018; Berriat et al. 2023; Weber et al. 2022; Zhong et al. 2017; Yang et al. 2020; Bandow et al. 2022; Winfree et al. 2022; Chang et al. ). However, further mechanistic studies integrating plasma, CSF, and cellular analyses are required to clarify the biological basis of these observations.

The strong influence of age identified in this study has important implications for the future clinical application of plasma sTREM2. Age‐adjusted reference intervals or age‐specific decision thresholds may be necessary to avoid misinterpretation of circulating concentrations, particularly in studies including elderly populations. Furthermore, the observation that plasma sTREM2 is reduced across different CNS disorders suggests that this biomarker may capture biological processes common to neurodegeneration and neuroinflammation rather than disease‐specific pathological pathways. Longitudinal studies will be essential to determine whether plasma sTREM2 predicts disease progression, cognitive decline, or therapeutic response. Interestingly, the Hisayama Study reported that higher serum sTREM2 concentrations were associated with an increased long‐term risk of incident dementia (Ohara et al. 2019), apparently contrasting with the reduced plasma levels observed in patients with established disease in our cohort. These findings, however, are not necessarily contradictory and may reflect differences in disease stage, study design, and analytical methodology. The Hisayama Study evaluated serum sTREM2 in cognitively normal individuals before the onset of dementia, whereas our study measured plasma sTREM2 in patients with established neurological disease, likely representing different phases of the disease continuum. In addition, differences in biological matrices and analytical platforms should be considered when comparing results across studies. Our previous work using the fully automated Lumipulse assay demonstrated a strong correlation between serum and plasma sTREM2 despite systematic differences in absolute concentrations, indicating that these matrices are closely related but not interchangeable (Agnello et al. 2026). Furthermore, the use of different analytical platforms, including ELISA‐based methods in earlier studies versus fully automated chemiluminescent immunoassays, may introduce methodological variability that contributes to discrepancies in reported sTREM2 concentrations. Collectively, these observations highlight the importance of considering both pre‐analytical and analytical factors when interpreting circulating sTREM2 across different clinical studies.

This study has several limitations. Its cross‐sectional design precludes assessment of longitudinal changes in plasma sTREM2 over the course of disease. Another limitation is the relatively small number of ATTR‐PN patients and asymptomatic carriers. This should be interpreted in the context of the rarity of hereditary transthyretin amyloidosis, for which the present cohort represents a comparatively large and well‐characterized series. Nonetheless, the possibility that modest differences in plasma sTREM2 remained undetected due to limited statistical power cannot be excluded, and confirmation in larger multicenter cohorts is warranted. Finally, the study was not designed to investigate the molecular mechanisms underlying the observed changes, and therefore, mechanistic interpretations remain speculative.

In conclusion, plasma sTREM2 follows a distinct age‐dependent trajectory in healthy individuals, characterized by an accelerated increase after approximately 68 years of age. This physiological pattern is significantly altered in ad, MCI, and MS but preserved in ATTR‐PN, highlighting disease‐specific differences in circulating sTREM2. Together, these findings indicate that plasma sTREM2 reflects biological processes distinct from those measured in CSF and emphasize the importance of considering both age and disease context when interpreting this biomarker. Future longitudinal and mechanistic studies will be essential to define the biological significance and clinical utility of circulating sTREM2.

## Author Contributions

**Giuseppe Salemi:** data curation. **Luisa Agnello:** conceptualization, writing – original draft, investigation. **Fabio Del Ben:** investigation. **Daniela Capello:** data curation. **Anna Masucci:** formal analysis. **Marcello Ciaccio:** conceptualization, methodology, validation, visualization, writing – review and editing, supervision. **Anna Maria Ciaccio:** writing – review and editing. **Vincenzo Di Stefano:** data curation. **Sabrina Novara:** formal analysis. **Caterina Maria Gambino:** formal analysis, writing – review and editing. **Tommaso Piccoli:** data curation.

## Funding

The authors have nothing to report.

## Ethics Statement

The study was conducted in accordance with the Declaration of Helsinki and approved by the Institutional Review Board of Policlinico “Paolo Giaccone,” Palermo, Italy (Approval No. 2/2026). Written informed consent was obtained from all participants prior to inclusion in the study.

## Conflicts of Interest

The authors declare no conflicts of interest.

## Data Availability

The data that support the findings of this study are available from the corresponding author upon reasonable request.
